# Correction: Simulation-based multiprofessional obstetric anaesthesia training conducted in situ versus off-site leads to similar individual and team outcomes: a randomised educational trial

**DOI:** 10.1136/bmjopen-2015-008344corr1

**Published:** 2017-11-08

Sørensen JL, van der Vleuten C, Rosthøj S, *et al*. Simulation-based multiprofessional obstetric anaesthesia training conducted in situ versus off-site leads to similar individual and team outcomes: a randomised educational trial. *BMJ Open* 2015;**5**:e008344. doi: 10.1136/bmjopen-2015-008344.

The table headings are incorrect on pages 2 and 3 of supplementary table S1. The sub-headings should read ‘OSS’ and ‘ISS’ - these headings are correct on page 1 of the Table but the wrong way round in pages 2 and 3.

10.1136/bmjopen-2015-008344corr1.supp1 Supplementary data

