# Supplementary material for: Correction: Simulation-based multiprofessional obstetric anaesthesia training conducted in situ versus off-site leads to similar individual and team outcomes: a randomised educational trial
Source: BMJ Open. 2017 Nov 8;7(11):e008344corr1. doi: 10.1136/bmjopen-2015-008344corr1 (PMC5695506; doi:10.1136/bmjopen-2015-008344corr1)
Supplement: Supplementary data [file bmjopen-2015-008344corr1supp001.pdf]

Sørensen JL et al, BMJ Open 2015: Simulation based multi-professional obstetric anaesthesia training conducted in situ versus off site leads to similar individual and team outcomes: a randomised educational trial.

Table S1. Suggestions for practical and organisational changes identified during ISS and OSS classified according to the model of work system or structure from the Systems Engineering Initiative for Patient Safety. [55]

	Identified during		SEIPS component
	OSS	ISS	
**Introduction of new employees**			
Better introduction of new employees, including guided tour of trauma centre, acute admission centre, and blood bank	X	X	Person
**Identification of staff**			
Name badges visible on uniforms at all times and improved name badges for easier identification of the various healthcare professions	X	X	Person
Inclusion of people’s names and also healthcare profession during staff presentation rounds in emergency situations	X	X	Organisation
**Call systems, telephones and telephone numbers**			
Pros and cons involved in changing the emergency call system from calling people individually to calling them as a group	X	X	Organisation
Request for more well-defined telephone chain for emergency calls; doctors preferred briefing to be from doctor to doctor	X	X	Organisation
More well-defined criteria for using emergency alarm button in delivery room	X	X	Organisation
Identical numbers to call night and day for anaesthesia assistance in obstetric emergencies	X	X	Organisation
A clearly visible list of relevant numbers in operating theatre for emergency situations		X	Tools & technology
Stickers with brief list of emergency numbers on back of name badges		X	Tools & technology
**Clinical handover in emergency situations**			
Repetition upon arrival in delivery room and operating theatre of clinical details and indication for procedures provided in telephone handover	X	X	Organisation
Consistent use of terminology from local guidelines and when grading emergency caesarean sections	X	X	Organisation
**Patient identification and “time out” in operating theatre in emergency situations**			
More clearly defined designation of who is responsible for identifying the patient and confirming the indication for procedure	X	X	Organisation
Improvement of computer system that is too difficult and slow for emergency situations		X	Tools & technology
**Presence of partners during emergency caesarean sections**			
Various opinions on whether partners should be allowed in operating theatre; more well-defined criteria for designating who communicates with partners	X	X	Organisation
**Medication – postpartum haemorrhage**			
Placement of tranexamic acid in the haemorrhage medication box; clinical guidelines on its administration should be made easily accessible	X	X	Tools & technology
Pre-prepared drips with oxytocin	X	X	Tools & technology
Midwives generally found administering medicine in operating theatre difficult; requested more clarity for designating who is responsible for the haemorrhage medication box there	X	X	Person, Task
Clarification of who is to document administration of medicine in operating theatre, especially when administered directly in the uterus and/or per rectum	X	X	Tools & technology

	Identified during		SEIPS components
	OSS	ISS	
**Medication – emergency caesarean sections**			
Pros and cons involved in administering terbutaline for tocolysis during foetal distress; informing anaesthesia staff important due to subsequent risk of maternal tachycardia	X	X	Tools & technology
Placement of terbutaline for tocolysis (intrauterine resuscitation) in delivery room and operating theatre to allow quick administration	X	X	Tools & technology
Address the knowledge gap among auxiliary nurses and midwives on administration of sodium citrate to prevent aspiration during general anaesthesia	X	X	Person, Task
Amend action card and clinical guidelines on emergency caesarean sections to specifically address oral administration of sodium citrate to prevent aspiration during general anaesthesia	X	X	Organisation
Make sodium citrate more easily accessible in the delivery room	X	X	Tools & technology
**Staff members in operating theatre during postpartum haemorrhage**			
Two operating theatre nurses ideally present in severe cases of postpartum haemorrhage	X	X	Person, Task
Obstetric team members (midwife, specialised midwife, and auxiliary nurse) should ideally prioritise remaining in operation theatre to assist	X	X	Person, Task
**Fluid resuscitation and blood transfusion in operating theatre**			
Clarification of whether lactated Ringer's solution is superior to sodium chloride	X	X	Tools + technology
Easy access to a blood heater and pressure bags to improve IV infusion in delivery room	X	X	Tools + technology
Clarification of which healthcare professional should ideally collect blood at the blood bank in an emergency situation	X	X	Person, Task
Clearly posted telephone number in operating theatre for the blood bank and its location	X	X	Tools + technology
Training/retraining of midwives in management of blood transfusions to allow them to assist the anaesthesia team correctly	X	X	Person, Task
**Transfer of patient from delivery room to operating theatre and type of operating table**			
Clarification of who is responsible for birthing bed (preparations for transport)		X	Person<br>Tools & technology
Mechanisms to ensure clear passage (e.g. no beds, transport cages) along corridors for emergency transport of patients on delivery ward		X	Person,<br>Tools & technology
Clarification of when and how to ideally transfer patients from delivery room to operating theatre; clarification of who determines timing of patient transport in emergency situations	X	X	Person,<br>Organisation
Improved standards for where to keep the remote control for the operating table and for recharging its batteries; have easy-to-use instructions available		X	Tools & technology
Pros and cons of continuous use of birthing beds in emergency situations when patient transferred to operating theatre; address the knowledge gap on functionality of birthing beds among operating theatre staff; establish standards for their use		X	Tools & technology
**Practical issues in operating theatre**			
Better labelling of equipment in operating theatre and standardised placement of equipment in the two operating theatres for obstetric emergencies to streamline management	X	X	Tools & technology
Improve use of remote control to the operation table, which is difficult due to a lack of clarity about which direction the table moves		X	Person,<br>Tools & technology
A more suitable walking distance between the cabinet with surgical caps and the operating theatre		X	Tools and technology

	Identified during		SEIPS components
	OSS	ISS	
**Operating theatre nurses**			
Clarification on role of responsibility for clinical decision making for urinary bladder catheter and/or shaving the pubic area, communication and actual management hereof; general agreement that obstetricians make the clinical decision and then inform the operating theatre nurse, who then places the catheter and/or shaves the area	X	X	Person, Organisation
**Anaesthesia team in operating theatre**			
More assistance from midwives when transferring patients from birthing bed to operating table and with positioning of patient; midwives requested more guidance from the anaesthesia team on the ideal way to do transfers	X	X	Person, Organisation
Improved procedures for checking equipment to ensure that it works (e.g. problem with no light in a laryngoscope)		X	Tools & technology, Organisation
More detailed introduction of new employees, including presentation of equipment for management of the difficult airway and equipment for blood heating and rapid infusion		X	Person
**Observation charts and boards**			
Improved observation charts for emergency situations, especially for postpartum haemorrhage	X	X	Tools & technology, Organisation
Greater use of white boards in delivery rooms in emergency situations for temporary observational charting	X	X	Tools & technology, Organisation
Use of white boards in operating theatre in emergency situations	X	X	Tools & technology, Organisation
**Mode of anaesthesia in emergency situations**			
Determine who makes final clinical decision about mode of anaesthesia; generally agreed to be the anaesthetist's responsibility	X	X	Person
Preoxygenation necessary prior to induction of spinal anaesthesia in case general anaesthesia is required	X	X	Task, Organisation
Clear communication on mode of anaesthesia to all staff in the room crucial so operating theatre nurses can prepare for e.g. sterile drapes, leg holders	X	X	Task, Organisation
Use, when feasible, obstetric manoeuvres like bimanual compression with severe postpartum haemorrhage and replacement of foetal head during cord prolapse with the parturient woman in side position (for attempt of spinal anaesthesia); communicate this during training/retraining of staff and address in clinical guidelines	X	X	Task, Organisation
**Guidelines**			
Greater clarity in postpartum haemorrhage guidelines on indications and general clinical management principles for blood product transfusion and risk of hypothermia	X	X	Task, Organisation
Addition of pointers in local clinical guidelines on how to choose the best team leader and this individual's role in emergency situations	X	X	Task, Organisation

ISS: in situ simulation; OSS: off site simulation; SEIPS: Systems Engineering Initiative for Patient Safety
